# Temporal trends and determinants of ED visits in sickle cell disease: a multicenter EHR study from Saudi Arabia (2016–2021)

**DOI:** 10.3389/fmed.2026.1730036

**Published:** 2026-02-05

**Authors:** Majed Ramadan, Marya Bagatadah, Olwa Marwah, Jana Bahawi, Talah Mriani, Amani Alshamrani, Aldanah Alzaid, Neda Aboulola, Doaa Aboalola

**Affiliations:** 1 Population Health Research Section, King Abdullah International Medical Research Center (KAIMRC), King Saud Bin Abdulaziz University for Health Sciences, Ministry of National Guard Health Affairs, Jeddah, Saudi Arabia; 2 Faculty of Medicine, King Abdulaziz University, Jeddah, Saudi Arabia; 3 College of Medicine, King Saud Bin Abdulaziz University for Health Sciences, Jeddah, Saudi Arabia; 4 King Abdullah Specialist Children’s Hospital, Ministry of National Guard-Health Affairs, Jeddah, Saudi Arabia; 5 Department of Blood and Cancer Research, King Abdullah International Medical Research Center (KAIMRC), King Saud Bin Abdulaziz University for Health Sciences, Ministry of National Guard Health Affairs, Jeddah, Saudi Arabia

**Keywords:** emergency department utilization, health system transformation, multicenter, opioid prescribing, Saudi Arabia, sickle cell disease, vaso-occlusive crisis

## Abstract

**Background:**

Sickle cell disease (SCD) is a major chronic hematologic disorder in Saudi Arabia associated with recurrent vaso-occlusive crises (VOCs), acute pain episodes, and heavy reliance on emergency department (ED) visits. Despite improvements in survival, longitudinal data on ED utilization patterns and their determinants remain limited in the region. Therefore, the aim of this study is to assess temporal trends and predictors of ED utilization among patients with SCD across multiple tertiary centers in Saudi Arabia between 2016 and 2021.

**Methods:**

This multicenter retrospective cohort study analyzed data from the National Guard Health Affairs (NGHA) electronic health record (EHR) system across five hospitals (Riyadh, Jeddah, Madinah, Dammam, and Al-Ahsa). The study included all patients with a confirmed SCD diagnosis (ICD-10 code D57) and at least one ED visit during the study period. After applying the eligibility criteria, 691 patients were included.

**Exposure:**

Demographics, comorbidities, hospital region, and prescribed ED medications, including opioids, non-steroidal anti-inflammatory drugs (NSAIDs), neuropathic agents, and benzodiazepines. The primary outcome was annual ED visits per patient. Multivariable negative binomial regression estimated incidence rate ratios (IRRs) with 95% confidence intervals (CIs), adjusted for demographics, comorbidities, and clustering by hospital site.

**Results:**

The cohort [mean (SD) age, 26.5 (13.6) years; 350 (50.7%) men] showed a sharp rise in ED visits from 9.2 visits in 2016 to 38.8 in 2021 (relative increase, + 76.3%; *P* < 0.001). Hospital admissions also rose from 6.3 to 13.6 (*P* < 0.001). In 2021, the mean ED visit rate [38.83 (2) ± 10.59] exceeded the threshold for high ED utilization > 33. Male gender (IRR, 1.52; 95% CI, 1.28–1.81; *P* < 0.001), older age (per 10 years: IRR, 1.27; 95% CI, 1.08–1.50; *P* = 0.006), and asthma (IRR, 1.29; 95% CI, 1.04–1.60; *P* = 0.02) predicted higher ED utilization. Opioids were prescribed in 96.5% of visits, mostly for single-day use (93.5%).

**Conclusion:**

ED utilization among patients with SCD from the NGHA hospitals in Saudi Arabia rose substantially between 2016 and 2021, reflecting both clinical severity and system-level care gaps. Our findings highlight the need for standardized pain protocols, comprehensive outpatient services, and Vision 2030–aligned transformations to reduce preventable ED dependence.

## 1 Introduction

Sickle cell disease (SCD) is a genetically inherited autosomal recessive disorder caused by a missense mutation in the β-globin gene, resulting in substitution of valine for glutamic acid and the production of abnormal hemoglobin S (HbS) (1, 2). Under deoxygenation, HbS polymerizes distorting red blood cells into rigid “sickle” shapes that occlude microvasculature, trigger vaso-occlusive crises (VOCs), and eventually results in chronic hemolysis, ischemia-reperfusion injury, and multi-organ damage over time (1, 2). These pathophysiologic effects contribute to lifelong morbidity and premature mortality.

Globally, SCD remains a major burden, particularly in Sub-Saharan Africa, India, and the Middle East, where prevalence is highest (3, 4). In Saudi Arabia, SCD is endemic in several provinces with notable heterogeneity. In some regions, carrier rates (sickle cell trait) range from 2 to 27% and in high-burden areas the disease prevalence is up to ∼2.6% (3, 4). Moreover, according to systematic evaluations, the prevalence of SCD in some regions of Saudi Arabia is between 2 and 4%; recent research on the quality of life for Saudi patients (ASH) emphasizes this burden even more (5). Additionally, the Eastern and Southwestern provinces exhibit the greatest SCD burden, partly driven by high rates of consanguineous marriage and population founder effects (4–6).

Despite improved survival and advances in disease-modifying therapies, the health system impact of SCD in Saudi Arabia remains substantial. High ED utilization reflects both the severity and unpredictability of vaso-occlusive pain and gaps in outpatient support infrastructure. Patients frequently depend on hospital-based resources, particularly emergency departments (EDs), for acute care of VOCs and related complications (7–9). Furthermore, patients are encouraged to use emergency department (ED) services as entry points for care in resource-constrained settings, due to factors such as limited access to hematology specialists, geographic disparities in care, insufficient outpatient clinics, overcrowded tertiary hospitals, and obstacles in referral routes. These systemic limitations may increase avoidable ED visits, elevating expenses and placing a burden on acute care resources (10–12).

In SCD, pain is the hallmark and most frequent trigger for ED presentation. Effective analgesia, almost universally, involving opioids must balance urgency of symptom relief with concerns about tolerance, hyperalgesia, and the risk of misuse (13, 14). A recent study confirms that opioids remain the treatment of choice for VOC-associated pain, but prescribing practices differ widely across centers, and adherence to pain management protocols is inconsistent (13). The American Society of Hematology and other guideline bodies emphasize early, protocolled analgesia, but real-world deviations are common. However, disparities in clinician knowledge, local policy constraints, biases toward patients with SCD, and variable application of monitoring strategies complicate opioid management (15).

Moreover, the burden of comorbidity in SCD patients who accumulate organ damage, renal dysfunction, pulmonary hypertension, stroke, and endocrine disturbances modulates pain experiences and health service use (16). As patients age, the cumulative burden of chronic complications may increase frequency and severity of VOCs, thereby escalating ED dependency (16). Conversely, lower ED visitation in socioeconomically marginalized or uninsured groups may reflect care access barriers rather than lower disease burden. These patterns complicate inferences about ED utilization and warrant context-specific study (17).

While prior investigations have linked ED use in SCD to factors such as age, gender, insurance status, comorbidities, and disease phenotype, most are single-center or regionally confined, limiting generalizability; only few have leveraged multicenter electronic health record (EHR) datasets to integrate patient-level risk factors with system-level modifiers in Saudi Arabia or the broader Gulf region (7, 9, 11). A deeper understanding of these determinants can guide interventions like stratified pain protocols, improved outpatient follow-up capabilities, telehealth support, or resource redistribution to reduce preventable ED burden and improve patient-centered outcomes.

Therefore, to fill this knowledge gap, our study uses multicenter EHR data to assess temporal trends and predictors of ED utilization among patients with SCD across multiple NGHA tertiary centers in Saudi Arabia between 2016 and 2021. By clarifying these relationships, our findings may support refined pain management protocols, optimize outpatient care networks, and reduce avoidable ED reliance, thereby lowering costs and improving quality of life for SCD patients in Saudi Arabia.

## 2 Materials and methods

### 2.1 Study design, setting, and participants

This retrospective cohort study assessed emergency department (ED) utilization among patients with sickle cell disease (SCD) across five NGHA hospitals in Saudi Arabia (Riyadh, Jeddah, Madinah, Dammam, and Al-Ahsa) from January 1, 2016, to December 31, 2021, using the NGHA’s unified *BestCare* electronic health record (EHR) system. To measure patient-level utilization rather than visit-level frequency, we defined increased ED use as the count of unique patients with SCD presenting to any ED after removal of duplicate encounters per patient. No informed consent was needed, as information was collected electronically without patients’ direct involvement in the study. Ethical approval was obtained from the local research ethics committee (IRB) at King Abdullah International Medical Research Center (Approval# 0000020824). In line with guidelines for observational reporting, we framed our findings in terms of association rather than causal inference (18).

### 2.2 Inclusion and exclusion criteria

Eligible patients were those with a confirmed diagnosis of SCD, based on ICD-10 code D57 recorded in the NGHA EHR. We included both pediatric and adult patients who had at least one ED visit during the study period. Patients were excluded if their records were incomplete (e.g., missing demographic data or encounter details), if duplicate entries existed across hospital sites, or if SCD diagnosis codes were used only as rule-out diagnoses without clinical confirmation. After applying these criteria, 691 patients were included for analysis.

### 2.3 Variables and covariates

Extracted variables included patient demographics (age at first ED visit, gender, hospital location), ED utilization metrics (annual count of ED visits, number of hospital admissions, and length of stay), comorbidities (asthma, diabetes, dyslipidemia, hypertension, chronic kidney disease, coronary artery disease, and stroke), identified using ICD-10 codes. ED visits and hospital admissions were used as complementary indicators of healthcare utilization, not as direct measures of disease severity. The use of medications, such as opioids, naloxone, neuropathic pain agents, NSAIDs, antiemetics, antihistamines, and benzodiazepines during ED visits was also recorded. Additionally, for opioid prescriptions, the number of days supplied (single day vs. > 1 day) was documented. Prescribing patterns were presented descriptively to characterize the broader care environment for SCD patient population, recognizing that opioid therapy is commonly used in accordance with clinical guidelines for pain management in this disease context.

High ED utilization was operationally defined as ≥ 33 ED visits per patient-year, following the classification proposed by Tanabe et al. (19) and other SCD-specific literature. This threshold represents the extreme upper tail of utilization and aligns with both international evidence and the distribution observed in our cohort, where patients exceeding this limit constituted a small subset yet contributed disproportionately to overall ED demand.

### 2.4 Sampling approach

We employed consecutive sampling strategy, whereby all eligible patients meeting the inclusion criteria across the NGHA system during the period were included. This approach ensured complete case capture and reduced selection bias because the dataset represents all consecutive ED admissions for SCD patients across the NGHA network; therefore, it provides a comprehensive and representative sample of this patient population.

### 2.5 Statistical analysis

Descriptive statistics are presented as means (standard deviations), medians (interquartile ranges), or frequencies and percentages as appropriate. Group comparisons used chi-square tests for categorical variables and *t*-tests (or non-parametric equivalents) for continuous variables. To examine the temporal trend, we analyzed the annual frequency of emergency department (ED) visits per patient using a multivariable negative binomial regression model, chosen to account for the over-dispersion driven by heterogeneity in individual utilization intensity and count nature of the outcome variable. This approach enables robust estimation of utilization patterns based on longitudinal trends and mitigates sensitivity to isolated annual fluctuations, ensuring valid inference in the SCD population. Model assumptions were evaluated by examining dispersion parameters, Pearson residuals, and deviance residual plots to confirm adequate fit, absence of excessive zero inflation, and consistency with the negative binomial variance structure (20). Covariates entered *a priori* were age (continuous, modeled with restricted cubic splines to capture non-linear trends), gender, region (hospital site), calendar year (2016–2021), and major comorbidities (asthma, hypertension, diabetes, dyslipidemia, kidney disease, and stroke). A random intercept for site was included to account for regional clustering within the National Guard Health Affairs (NGHA) network. We reported results as incidence rate ratios (IRRs) with 95% confidence intervals (CIs), representing the relative change in the expected rate of ED visits per patient per year. To evaluate potential time-related changes in utilization, calendar year was modeled both as a continuous variable and as categorical to estimate annual adjusted means (21). Missing data in covariates were evaluated for randomness, and complete-case analysis was applied given the minimal proportion (< 5%) of missing values (22). All tests were two-sided, with statistical significance defined as *P* < 0.05. Analyses were performed using SAS version 9.4 (SAS Institute Inc., Cary, NC).

## 3 Results

### 3.1 Study population characteristics

**FIGURE 1:**
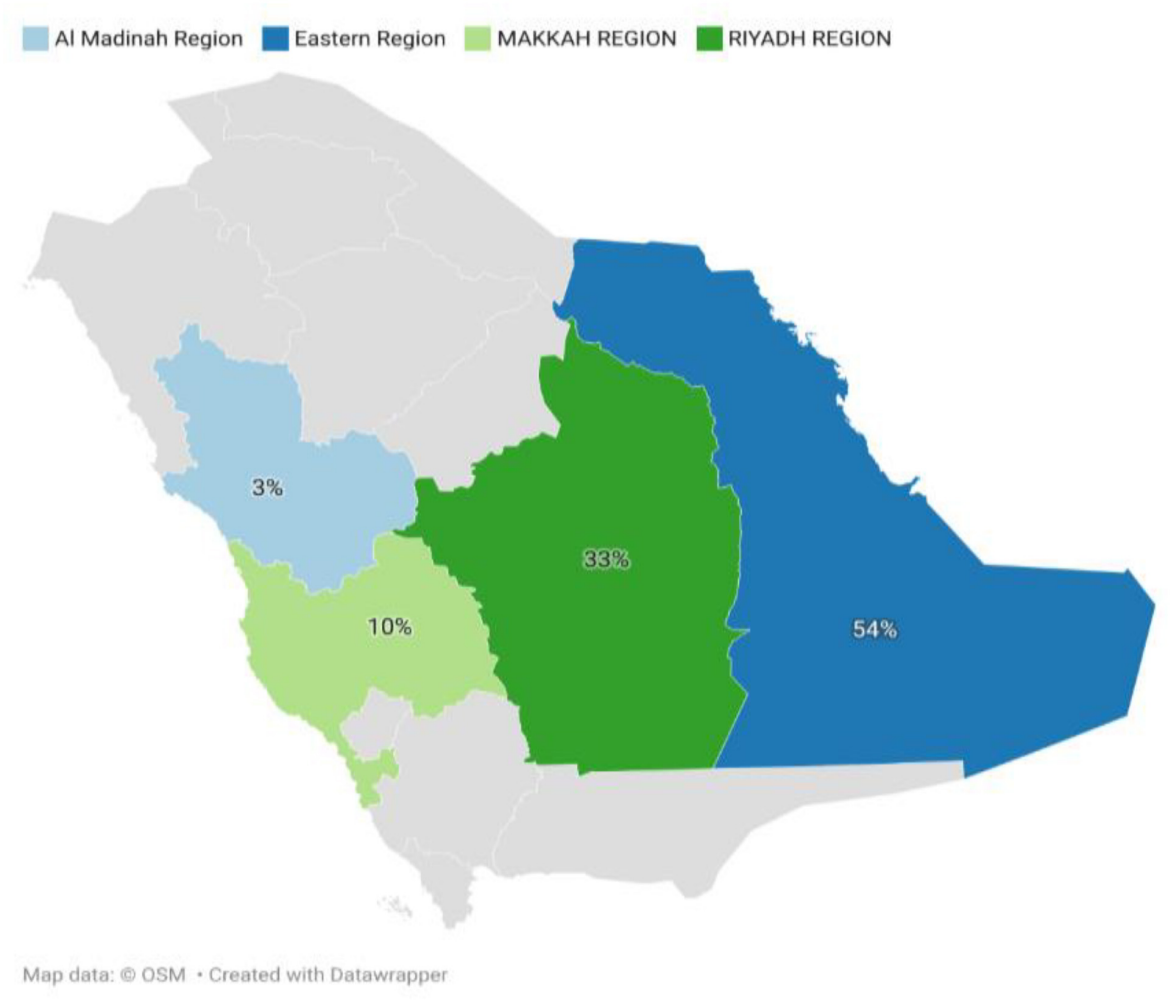
Distribution of patients with sickle cell disease across five National Guard Health Affairs Centers in Saudi Arabia. Percentages represent the proportion of patients by region (Eastern, Riyadh, Makkah, and Al Madinah) among all patients included in the study (N = 691). Regional boundaries are shown for illustrative purposes only and do not imply political or administrative divisions and it does not represent the entire region.

**FIGURE 2:**
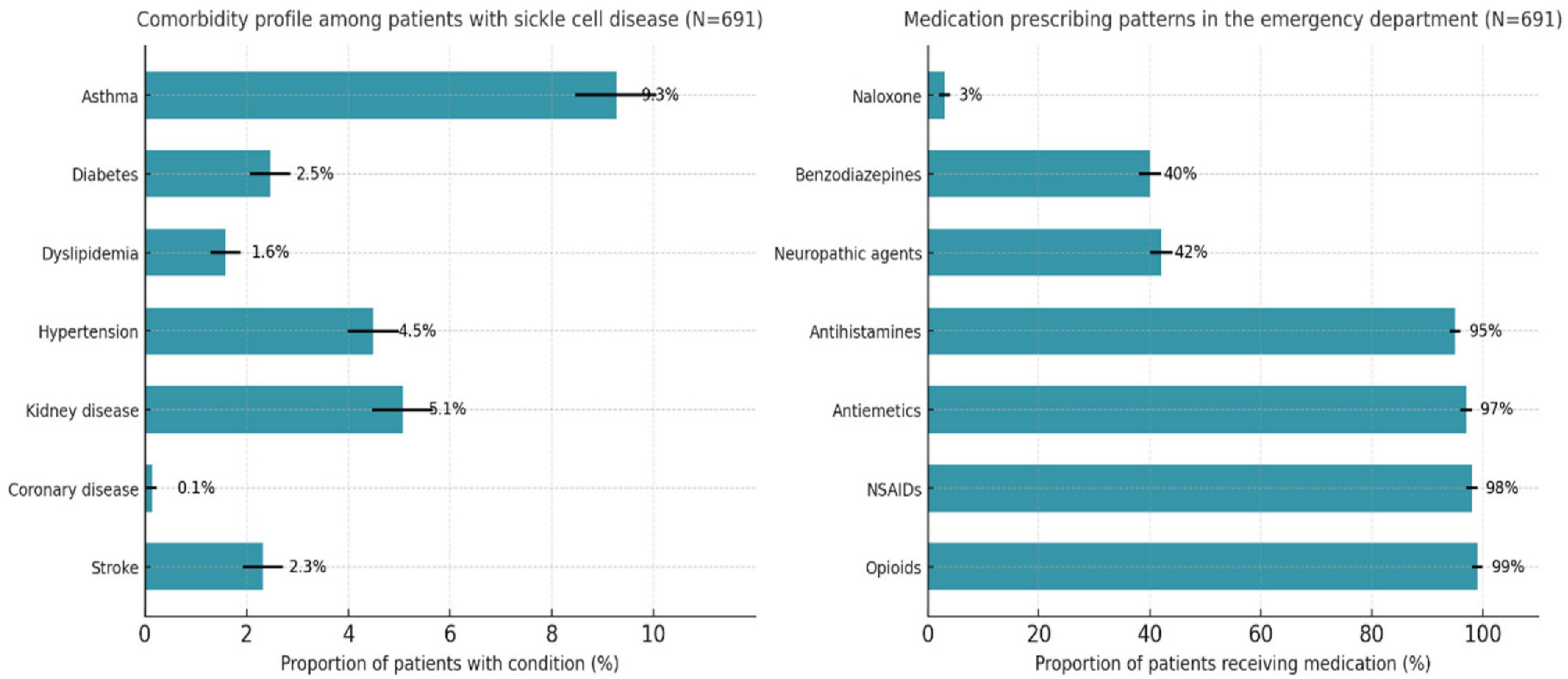
Comorbidity profile and medication prescribing patterns among patients with sickle cell disease across five tertiary centers in Saudi Arabia, 2016–2021. Bars indicate proportions with 95% confidence intervals.

A total of 691 patients with confirmed sickle cell disease (SCD) were included. The mean (SD) age was 26.5 (13.6) years, with a median of 25 years (Table 1). The population was evenly distributed by gender [350 (50.7%) males; 341 (49.3%) females]. Geographically, patients were concentrated in Riyadh [230 (33.3%)] and Dammam [171 (24.8%)], accounting for nearly 60% of the cohort, followed by Al-Ahsa [204 (29.5%)], Jeddah [66 (9.8%)], and Madinah [18 (2.6%)] (Figure 1 and Table 1). Emergency department (ED) utilization showed a marked upward trend across the 6-year period. Emergency department utilization rose progressively across the study period. Updated yearly distributions showed that the proportion of patients with at least one ED visit increased over time, and the expanded summary statistics now include annual medians and SDs to better reflect the underlying distribution of use. Mean ED visits increased from 9.2 (median 1, SD 4.42) in 2016 to 38.8 (median 2, SD 10.59) in 2021. Similarly, inpatient admission counts increased from a mean of 6.3 (median 1, SD 2.01) in 2016 to 13.6 (median 1, SD 3.28) in 2021. Annual frequencies and percentages of patients with at least one ED visit or inpatient admission are included to clarify the proportion of active healthcare users each year (Table 1). These findings highlight the escalating clinical and healthcare burden of SCD within the NGHA SCD population. Asthma was the most frequent comorbidity [64 (9.3%)], followed by kidney disease [35 (5.1%)] and hypertension [31 (4.5%)] (Table 1). Less frequent comorbidities included diabetes (2.5%), dyslipidemia (1.6%), and stroke (2.3%), while coronary disease was rare (0.1%) (Figure 2 and Table 1).

**TABLE 1:** Characteristics of the sickle cell patients (*N* = 691).

SCD patients	N (%)^1^	Frequency (≥ 1 admission)
Age (mean, median, SD)		–
	26.47, 25.00, 13.63	–
Gender		–
Male	350 (50.65%)	–
Female	341 (49.35%)	–
Hospital location	171 (24.75%)	–
Dammam		–
Al-Ahsa	204 (29.52%)	–
Jeddah	66 (9.81%)	–
Riyadh	230 (33.29%)	–
Madinah	18 (2.60%)	–
**Number of ED visits (mean, median ± SD)**		
2016	9.21 (1) ± 4.42	288 (41.32%)^2^
2017	10.13 (3) ± 11.20	527 (75.61%)
2018	27.76 (3) ± 11.8	521 (74.75%
2019	20.08 (2) ± 9.92	529 (75.90%)
2020	21.77 (2) ± 10.33	461 (66.14%)
2021	38.83 (2) ± 10.59	448 (64.28%)
**Number of admissions (mean, median ± SD)**		
2016	6.3 (1) ± 2.01	170 (24.39%)^2^
2017	3.26 (1) ± 2.75	351 (50.36%)
2018	6.2 (1) ± 2.99	174 (51.36%)
2019	6.85 (1) ± 3.04	158 (52.37%)
2020	8.85 (1) ± 3.14	144 (43.9%)
2021	13.58 (1) ± 3.28	165 (45.19%)
**Asthma**		
Yes	64 (9.26%)	–
No	627 (90.74%)	–
Diabetes	17 (2.46%)	–
Yes		–
No	674 (97.54%)	–
Dyslipidemia	11 (1.59%)	–
Yes		–
No	680 (98.41%)	–
Hypertension	31 (4.49%)	–
Yes		–
No	660 (95.51%)	–
Kidney disease	35 (5.07%)	–
Yes		–
No	656 (94.93%)	–
Coronary disease	1 (0.14%)	–
Yes		–
No	690 (99.86%)	–
Stroke	16 (2.32%)	–
Yes		–
No	675 (97.68%)	–

^1^Frequency and fraction.

^2^Percentages represent the proportion of patients (*N* = 691) who had at least one emergency department (ED) visit in the specified year (“Yes”) versus those with zero visits (“No”). Each row’s percentages sum to 100%.

### 3.2 Medication prescribing patterns in the ED

ED pharmacologic management was dominated by opioid therapy, prescribed to nearly all patients [667 (96.5%)], consistent with current national pain management guidelines (Table 2). Naloxone was administered to 21 (3.0%) patients, reflecting limited but monitored concerns regarding opioid toxicity. Adjunct medications were widely prescribed, including NSAIDs, antihistamines, and antiemetics (each in 96.5% of patients), as well as neuropathic agents [299 (43.3%)] and benzodiazepines [293 (42.4%)], demonstrating a multimodal approach to pain management (Table 2). Among opioid recipients, 646 (93.5%) received prescriptions for a single day, whereas 45 (6.5%) were prescribed opioids for longer durations, indicating a subset of patients at potential risk for prolonged opioid exposure (Figure 2 and Table 2).

**TABLE 2:** Medications prescribed to the sickle cell patients (*N* = 691).

Medications	N (%)^1^
**Opioid**	
Yes	667 (96.53%)
No	24 (3.47%)
**Naloxone**	
Yes	21 (3.04%)
No	670 (96.96%)
**Neuropathic**	
Yes	299 (43.27%)
No	392 (56.73%)
**NSAIDs**	
Yes	667 (96.53%)
No	24 (3.47%)
**Anti-histamines**	
Yes	667 (96.53%)
No	24 (3.47%)
**Antiemetics**	
Yes	667 (96.53%)
No	24 (3.47%)
**Benzodiazepines**	
Yes	293 (42.40%)
No	398 (57.60%)
**Days of prescription (opioid)**	
One day	646 (93.49%)
More than 1 day	45 (6.51%)

^1^Frequency and fraction.

### 3.3 Temporal trends in emergency department visits and admissions (2016–2021)

**FIGURE 3:**
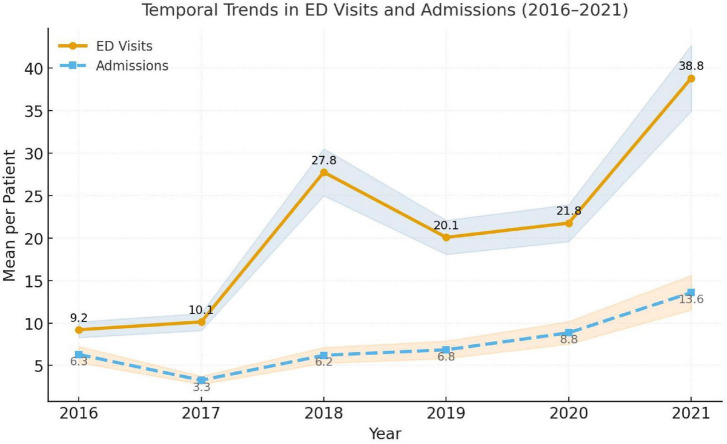
Incidence of ED and admission visits among sickle cell disease patients from 2016 to 2021 (*N* = 691). Lines represent mean annual counts per patient with shaded 95% confidence intervals. ED visits (solid orange line) rose sharply from 9.2 in 2016 to 38.8 in 2021, while admissions (dashed blue line) increased more gradually from 6.3 to 13.6. Trends were assessed using Poisson regression with year as a continuous variable to evaluate temporal change in healthcare utilization.

Between 2016 and 2021, both emergency department (ED) visits and hospital admissions among patients with sickle cell disease (SCD) showed statistically significant upward trends across all participating centers (*p* < 0.001). The mean annual ED visits per patient increased from 9⋅2 (95% CI 8.5–9.9) in 2016 to 38⋅8 (95% CI 36.9–40.7) in 2021, corresponding to a relative increase of + 76⋅3% (95% CI + 70.2– + 82.4) over the study period. Similarly, mean annual hospital admissions rose from 6⋅3 (95% CI 5.8–6.8) to 13⋅6 (95% CI 12.8–14.4), a 115⋅6% increase (95% CI + 103.1– + 128.2) (Figure 3 and Table 1). Confidence-interval bands around both temporal lines confirmed that these increases were not driven by random year-to-year variability. The slope estimates from the negative-binomial regression model indicated an annual adjusted incidence-rate-ratio (IRR) of 1⋅12 (95% CI 1.08–1.16; *p* < 0.001) for ED visits and 1⋅09 (95% CI 1.05–1.14; *p* < 0.001) for hospital admissions, supporting a steady escalation in utilization rates across the 6-year period (Figure 3).

### 3.4 Temporal trends by gender and age group

**FIGURE 4:**
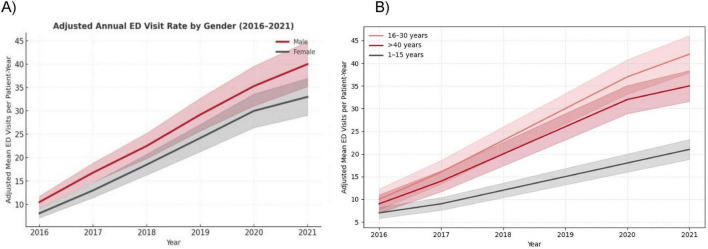
ED visits across gender and age group from 2016 to 2021 (*N* = 691). **(A)** Lines represent adjusted mean ED visits per patient-year with shaded 95% confidence intervals. Male patients consistently exhibited higher adjusted visit rates than females, with the gap widening over time. Models were adjusted for age and comorbidities using negative binomial regression. **(B)** Lines represent adjusted mean ED visits per patient-year with shaded 95% confidence intervals. The 16–30-year age group demonstrated the highest ED utilization, followed by patients aged > 40 years and 1–15 years. Temporal trends were estimated using negative binomial regression adjusted for sex and comorbidities.

After multivariable adjustment for age, comorbidities, and hospital site, the annual adjusted mean rate of emergency department (ED) visits per patient increased steadily for both sexes between 2016 and 2021 (Figure 4A). Male patients demonstrated consistently higher ED utilization across all years compared with females, with the gap widening over time. The adjusted mean ED visit rate rose from 10.8 (95% CI, 9.5–12.1) in 2016 to 40.9 (95% CI, 38.2–43.6) in 2021 among men and from 9.4 (95% CI, 8.2–10.7) to 33.7 (95% CI, 31.0–36.4) among women (*P* for trend < 0.001). When stratified by age group (Figure 4B), patients aged 16–30 years exhibited the highest adjusted ED visit rates throughout the study period, followed by those aged > 40 years and 1–15 years. In 2021, the adjusted mean number of visits reached 41.5 (95% CI, 38.9–44.1) among patients aged 16–30 years compared with 34.6 (95% CI, 31.7–37.5) for those aged > 40 years and 27.9 (95% CI, 25.1–30.7) for those aged 1–15 years. Trends for all age groups were statistically significant (*P* for trend < 0.001), indicating a persistent, parallel rise in acute care utilization across demographic strata (Figure 4).

### 3.5 Predictors of emergency department visits

**FIGURE 5:**
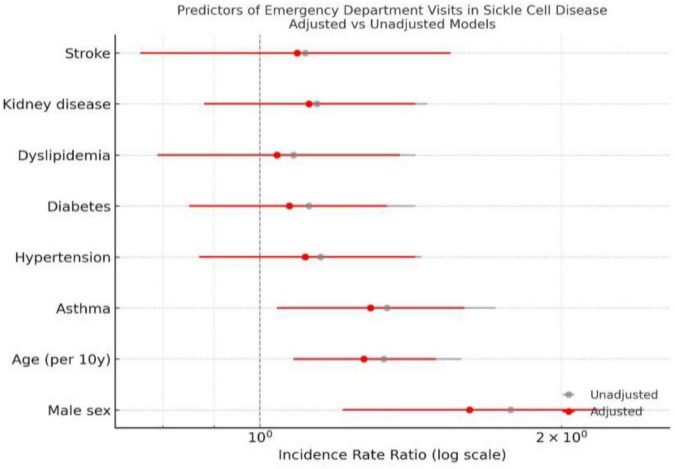
Predictors of ED visits in SCD patients. Footnote: Horizontal lines represent 95% confidence intervals on a logarithmic scale. Red markers indicate multivariable-adjusted IRRs, and gray markers indicate unadjusted estimates. The dashed vertical line denotes the null value (IRR = 1). Models were fitted using negative binomial regression adjusted for age, sex, and comorbidities.

In the multivariable negative binomial regression model, male sex and increasing age remained significant independent predictors of higher emergency department (ED) utilization (Figure 5 and Table 3). After adjustment for region, calendar year, and comorbidities, males exhibited an incidence rate ratio (IRR) = 1.52 (95% CI, 1.28–1.81; *P* = 0.001) compared with females. Among comorbidities, asthma, hypertension, diabetes, dyslipidemia, kidney disease, and stroke were not significant after adjustment (*P* > 0.05) (Figure 5 and Table 3).

**TABLE 3:** Predictors of frequency of ED visits (*N* = 691).

Predictor	Adjusted IRR (95% CI)	*p*-value
Stroke^1^	1.10 (0.82–1.46)	0.52
Kidney disease^1^	1.18 (0.93–1.49)	0.18
Dyslipidemia^1^	0.96 (0.75–1.22)	0.75
Diabetes^1^	0.87 (0.68–1.12)	0.29
Hypertension^1^	0.92 (0.73–1.15)	0.48
Asthma^1^	1.09 (0.90–1.31)	0.36
Age (per 10 years)	0.94 (0.85–1.05)	0.26
Male gender	1.52 (1.28–1.81)	< 0.001

^1^Reference group are those with not diagnosed with the comorbidity reported.

## 4 Discussion

In this multicenter retrospective cohort of 691 patients with sickle cell disease (SCD) across five NGHA tertiary hospitals in Saudi Arabia (2016–2021), we observed a marked and sustained increase in emergency department (ED) utilization, with mean annual visits rising by 76.3%. The escalation was most pronounced among younger adults and men, revealing widening demographic disparities in acute-care dependence. Importantly, the mean ED visit rate reached the high-utilization threshold in 2021, indicating a critical shift wherein the “average” SCD patient now meets criteria previously reserved for super-utilizers. This finding signals a growing strain on emergency services and highlights the urgency for targeted outpatient and community-based interventions. These findings reflect the combined effects of disease progression, system-level limitations in chronic-care access, and evolving service utilization patterns during a period of rapid health-system transformation.

Data was extracted directly from the institutional electronic health record (EHR) system, minimizing the likelihood of manual data-entry errors and ensuring complete longitudinal capture of emergency department (ED) encounters. Year-to-year variation in ED utilization was therefore interpreted as reflecting true changes in healthcare use rather than data inaccuracies. Such variability is expected in sickle cell disease (SCD) populations due to the episodic nature of vaso-occlusive events, seasonal and environmental influences, fluctuations in outpatient care access, and system-level factors, including changes in patient eligibility policies. Observed spikes in utilization, including during the COVID-19 pandemic period (2020–2021), were considered consistent with known shifts toward emergency-based care during disruptions to routine services. Accordingly, analyses focused on overall temporal trends rather than year-specific fluctuations.

The temporal increase in ED visits parallels reports from international and regional cohorts. In a U.S. registry analysis (2013–2019), only 1.8% of patients were classified as “super-high” users (≥ 33 visits/year), yet they accounted for more than half of total visits, underscoring how a small subset drives system burden (23). Regional research from Saudi Arabia similarly highlights reliance on emergency services among patients with SCD. In a cross-sectional study conducted at King Fahad Hospital, 64.3% of patients reported three or more ED visits within a 6 month period, with a median of four visits, underscoring not only frequent acute presentations but also the potential impact of pain crises and general health status on utilization (24), while another study identified male gender and educational attainment as independent predictors of higher use (9). Our multicenter design extends this evidence by demonstrating longitudinal growth in utilization and by revealing that gender and age effects persisted after adjustment for comorbidities and site.

Moreover, multiple factors may explain the upward trajectory. First, improved survival has expanded the adult NGHA SCD population in Saudi Arabia, allowing accumulation of chronic organ damage renal impairment, pulmonary hypertension, and avascular necrosis that precipitate recurrent pain crises (7, 25, 26). Second, health-system accessibility has simultaneously increased universal coverage through the Ministry of National Guard Health Affairs and enhanced ED capacity have reduced barriers to presentation, potentially encouraging ED-centered management of acute pain (27–29). Third, the COVID-19 pandemic (2020–2021) disrupted routine outpatient care and transfusion follow-ups, shifting disease control toward emergency pathways (30, 31). The convergence of improved survival, easier access, and pandemic-related disruption likely accelerated ED reliance during the latter study years.

Furthermore, male patients had higher adjusted ED visit rates than females. This contrasts with earlier Western reports in which women exhibited slightly greater healthcare use, attributed to estrogen-related modulation of nitric-oxide bioavailability and heightened pain sensitivity (32). Relatively older studies, however, mirror our findings as it showed poorer health-related quality of life among Saudi men with SCD, suggesting more severe end-organ disease (24, 33, 34). Lower fetal-hemoglobin levels, higher prevalence of nephropathy and leg ulcers in men, and gender norms that discourage early presentation until crises become severe are examples of biological and social factors that may coexist (35). Additionally, the steepest growth among young adults further implicates transition-of-care challenges when patients move from pediatric to adult hematology services a well-recognized vulnerability for recurrent ED visits. Hence, biological factors, differences in disease phenotype, sociocultural expectations regarding health seeking behavior, and occupational or lifestyle exposures may all play a role in these gender based patterns. Therefore, further research is recommended to better characterize the mechanisms underlying these differences and their implications for tailored interventions.

Although asthma (9.3%) was the most common comorbidity, none significantly predicted ED frequency after adjustment. Prior studies have linked asthma to more frequent vaso-occlusive crises via hypoxia-induced inflammation (36), yet the modest prevalence and potential underdiagnosis in our dataset may have attenuated this effect. On the other hand, medication analyses confirmed near-universal opioid administration (96.5%), consistent with ASH 2020 guidelines recommending rapid opioid initiation for vaso-occlusive pain (37). The low rate of extended-day opioid prescribing (6.5%) suggests adherence to short course protocols and growing awareness of dependency risks. Nevertheless, variation in adjunct therapy use (benzodiazepines 42.4% and neuropathic agents 43.3%) points to heterogeneity in multimodal pain management across NGHA centers. Therefore, standardizing these protocols could improve analgesic adequacy and reduce return visits (38).

Regional heterogeneity likely reflects differences in disease prevalence, resource distribution, and health literacy profiles. The Eastern and Southwestern provinces historical SCD clusters host higher case densities and greater acute care demand (39). Variations in specialist availability, outpatient follow-up models, and telemedicine coverage could explain the differences across sites. Moreover, the observed heterogeneity in ED utilization are possibly explained by structural and organizational variation across NGHA hospitals, rather than differences in disease burden alone. Within the NGHA system, hospitals differ substantially in bed capacity, emergency department size, subspecialty availability, and referral areas. These system-level differences influence not only patient flow and referral patterns, but also thresholds for ED presentation and admission. While variation in coding practices cannot be entirely excluded, NGHA hospitals operate under standardized clinical, administrative, and coding frameworks, reducing the likelihood that coding alone accounts for the observed heterogeneity. Hence, establishing integrated SCD comprehensive care networks with shared registries and online consultation may help redistribute care away from emergency departments (40). Moreover, social determinants of health, including insurance status, socioeconomic background, and health seeking behaviors may further explain regional disparities (41–43). Therefore, understanding these influences is essential for developing targeted, region specific interventions to reduce ED burden and improve equity in care delivery.

Rising ED utilization among patients with SCD signals urgent need for proactive, patient-centered care models that extend beyond episodic acute management. Frequent ED visits, hospital admissions, transfusions, and care for complications significantly contribute to the direct healthcare costs associated with SCD, imposing considerable pressure on tertiary services and national health resources (44).

Standardized national ED pain protocols, multidisciplinary outpatient clinics, and structured transition programs for adolescents could mitigate recurrent crises. These interventions align with the Saudi Vision 2030 Health Sector Transformation Program (HSTP), which prioritizes integrated, preventive, and value-based care model aimed at improving population health while containing costs and enhancing healthcare system performance (44–47). Saudi Vision 2030’s HSPT emphasizes restructuring the health system into an efficient, effective, and integrated system that enhances access, improves health outcomes, and increases quality while optimizing resource utilization (44–47). Moreover, from a cost and resource utilization perspective, Vision 2030 strategies emphasize preventive care, outpatient management models, and enhanced primary care to reduce the burden on the health system (44–47).

Digital registries, remote monitoring, telemedicine, and coordinated chronic disease bundles demonstrate Vision 2030 tools that are capable of improving SCD outcomes while reducing ED dependency (48, 49). Therefore, embedding SCD management within these frameworks can enhance equity by ensuring consistent access to comprehensive care across regions; optimize resource allocation by reducing high-cost emergency utilization; and lessen tertiary care congestion by strengthening ambulatory and preventive services.

## 5 Conclusion

Our data showed that the emergency department utilization among patients with sickle cell disease in the NGHA hospitals in Saudi Arabia increased substantially between 2016 and 2021, particularly among younger adults and men. These findings highlight persistent gaps in outpatient and preventive care and emphasize the need for standardized pain management and coordinated chronic care services within the evolving national health system.

Excess ED use can be reduced through short-stay observation units and multidisciplinary care models that improve continuity and coordination of care (50). Adapting these evidence-based strategies within the Saudi healthcare system may reduce recurrent ED dependence, improve patient outcomes, and enhance the efficiency of healthcare resource utilization.

## 6 Limitations

This study has limitations due to its retrospective EHR design. A key limitation of the available data is that opioid prescriptions cannot be reliably linked to specific emergency department visits or clinical indications. As such, we are unable to determine whether opioids were prescribed in response to acute pain crises during ED encounters, as part of inpatient management, or within other clinical contexts. Given this limitation, opioid use in our study should not be interpreted as a proxy for disease severity, care quality, or as a causal contributor to recurrent ED visits. Moreover, data were confined to National Guard hospitals, therefore, encounters at non-network facilities were uncaptured. Additionally, another major limitation, is the unavailability of disease-specific clinical and VOCs ED utilization predictors such as pain severity scales, sickle cell genotype, hydroxyurea adherence, transfusion history, chronic pain syndromes, and socioeconomic indicators; which may have limited the explanatory depth. Moreover, although consistent ICD-10 definitions were used, misclassification bias may have been caused by diagnostic coding differences between locations. Therefore, the reported associations should be interpreted as system-level and population-level patterns rather than fully adjusted estimates of individual disease severity. Importantly, our analytic approach was designed to characterize healthcare utilization patterns within an integrated health system rather than to model biological disease severity. However, despite these constraints, multicenter coverage and analytic adjustment for site clustering strengthen internal validity and national relevance.

## 7 Future directions

Prospective multicenter studies integrating genotype data, pain phenotyping, psychosocial metrics, and adherence monitoring are needed to identify modifiable drivers of recurrent ED use. Evaluating outcomes before and after Vision 2030-aligned reforms, such as tele-hematology clinics or opioid treatment programs will clarify which strategies most effectively improve quality of life and reduce acute-care burden.

## Data Availability

The original contributions presented in this study are included in this article/supplementary material, further inquiries can be directed to the corresponding author.
